# *CCNO* mutations in NPH?

**DOI:** 10.18632/aging.101379

**Published:** 2018-02-03

**Authors:** Marc Núnez-Ollé, Travis H Stracker, Gabriel Gil-Gómez

**Affiliations:** 1 Apoptosis Signalling Group, IMIM (Institut Hospital del Mar d’Investigacions Mèdiques), 08003 Barcelona, Spain

**Keywords:** CCNO, ciliogenesis, normal pressure hydrocephalus, neurodegeneration

Primary Ciliary Dyskinesia-29 (CILD29, OMIM#615872) is an autosomal recessive disorder characterized by early onset, progressive and irreversible lung damage due to recurrent respiratory infections caused by the inability of the multiciliated cells (MCC) present in the respiratory epithelium to clear mucus and particles trapped in the upper airways. The airways of these patients show a nearly complete lack of cilia on the MCCs or have a few dysfunctional, motile cilia. The molecular cause of CILD29 is mutations in the coding region of the *CCNO* gene, encoding an atypical cyclin necessary for the generation of multiple motile cilia [1]. Collectively, patients suffering from congenital mucociliary clearance disorders due to ciliary defects arising as a consequence of mutations in *CCNO* or other genes required to generate MCCs such as *MCIDAS* [2], are grouped under the term Reduced Generation of Multiple Motile Cilia (RGMC), a rare type of Primary Ciliary Dyskinesia (OMIM#244400).

In addition to the respiratory airways, MCCs are present in the male and female reproductive epithelium and in the ependyma, the epithelium lining the brain ventricles, accomplishing different functions depending on their location. The mouse oviduct coils and the human Fallopian tube plicae are covered by a single layer of multiciliated columnar epithelium interspersed with non-ciliated, secretory cells. This structure allows the propulsion of gametes and embryos and supports fertilization and early embryogenesis. We recently described that the complete lack of CCNO in the mouse results in male and female infertility [3].

In the ependyma, the MCCs are responsible for recirculating the cerebrospinal fluid (CSF). CSF is produced from blood plasma in the choroid plexuses within the brain ventricles and it is reabsorbed in the arachnoid granulations through the superior sagittal sinus and into the venous circulation. Defective recircularization of the CSF leads to its accumulation and the dilatation of the brain ventricles, a condition called hydrocephalus. Hydrocephalus can arise during the embryonic development (congenital) or can be acquired postnatally. Most of these patients show increased intracranial pressure and often need urgent intervention to drain the CSF. The lack of cilia or the presence of immotile cilia in the ependymal MCCs is one of the causes of congenital hydrocephalus. Constitutive *Ccno^-/-^* mice present few, abnormal cilia in the ependymal MCCs and develop hydrocephalus with higher penetrance than reported for the conditional knockout model or in the RGMC patients [1,3–5]. Hence, about 70% of the *Ccno*^-/-^ mice develop severe communicating hydrocephalus within the first month of postnatal life. Mice surviving this period can live for as long as their *Ccno^+/+^* or *Ccno^+/-^* siblings without overt neurological defects most likely due to compensation of the increasing intracranial pressure by thinning of the brain parenchyma.

Normal Pressure Hydrocephalus (NPH) is a form of communicating hydrocephalus of unknown molecular cause that can progress over decades without severe neurological manifestations. The prevalence of idiopathic NPH in the elderly is estimated to be up to of 3% in patients greater than 65 years old. However, the insidious onset of symptoms and the lack of acute episodes lead to hydrocephalus often being mistaken for other chronic neurodegenerative diseases such as Parkinson’s and Alzheimer’s diseases. Because of the lack of consistent clinical symptoms, it is very likely that the number of diagnosed patients is greatly underestimated [6].

**Figure 1:**
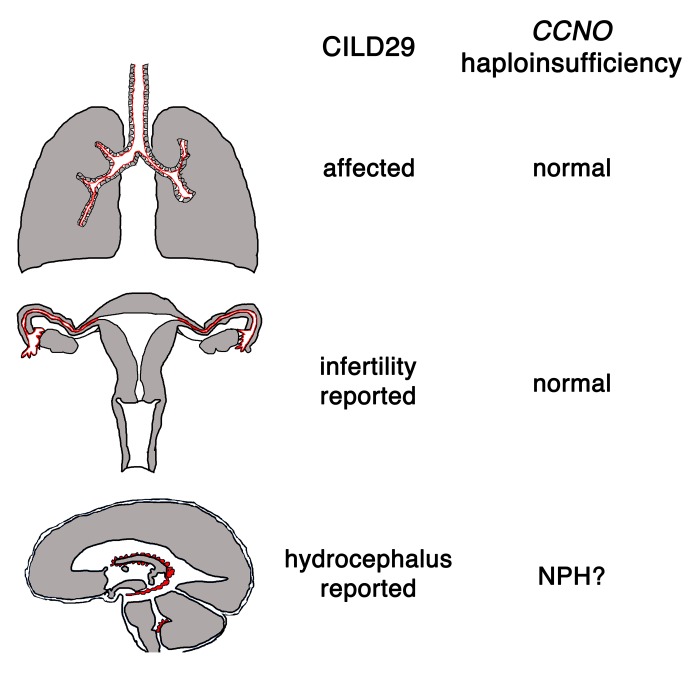
**Consequences of complete or partial loss of CCNO**. CCNO is involved in the generation of motile cilia in the MCCs of the respiratory airways, the Fallopian tubes and in the ependymal cells. CCNO mutations result in CILD29, a human syndrome characterized by the destruction of the lung parenchyma, female infertility and hydrocephalus. Most *Ccno^-/-^* mice and some *Ccno^+/-^* siblings develop communicating hydrocephalus. *CCNO* heterozygotes or carriers of hypomorphic mutations could constitute the molecular basis for idiopathic Normal Pressure Hydrocephalus (NPH). Epithelia containing MCCs are indicated with a red line.

Because of the similarities between the CNS phenotype of constitutive *Ccno^-/-^* mice and the physiopathological findings in human NPH patients, it is tempting to speculate that CCNO haploinsufficiency could represent a molecular cause for idiopathic NPH. Interestingly,

*Ccno^+/-^* mice also develop hydrocephalus, albeit with lower penetrance [3]. It is possible that the threshold levels of CCNO necessary for proper MCC development are variable, depending on the tissue type. If this is the case, reduced doses of CCNO, due to heterozygosity or to the existence of hypomorphic alleles, may result in patients suffering only some of the pathologies present in CILD29. Accordingly, full expression of the protein would be necessary for the correct development and function of MCCs in all tissues and its loss would result in the pathologies associated with CILD29. On the other hand, reduced CCNO levels present in heterozygous or hypomorphic carriers could allow for the correct development of the MCCs in some tissues, for example the airways and reproductive epithelia, while leading to an impaired development of the ependymal MCCs, with reduced penetrance, resulting in NPH (Figure 1).

Our results therefore suggest that the identification and follow up of heterozygous *CCNO* mutation carriers could be important as they may be at risk of developing NPH, a disease which has an effective treatment but is often misdiagnosed as other incurable and devastating neurodegenerative disorders.
